# A268 1 YEAR FOLLOW-UP OF PEDIATRIC ULCERATIVE COLITIS USING INTESTINAL ULTRASOUND

**DOI:** 10.1093/jcag/gwad061.268

**Published:** 2024-02-14

**Authors:** S Thornton, D Isaac, A Hudson, D MacKay, A Kuc, M W Carroll, E Wine, H Huynh

**Affiliations:** University of Alberta Faculty of Medicine & Dentistry, Edmonton, AB, Canada; University of Alberta Faculty of Medicine & Dentistry, Edmonton, AB, Canada; University of Alberta Faculty of Medicine & Dentistry, Edmonton, AB, Canada; University of Alberta Faculty of Medicine & Dentistry, Edmonton, AB, Canada; University of Alberta Faculty of Medicine & Dentistry, Edmonton, AB, Canada; University of Alberta Faculty of Medicine & Dentistry, Edmonton, AB, Canada; University of Alberta Faculty of Medicine & Dentistry, Edmonton, AB, Canada; University of Alberta Faculty of Medicine & Dentistry, Edmonton, AB, Canada

## Abstract

**Background:**

Intestinal ultrasound (IUS) is an emerging tool that offers a non-invasive method for monitoring inflammatory bowel disease in pediatric patients, with increasing popularity worldwide. Assessment of IUS for monitoring pediatric Ulcerative Colitis (UC) has been previously limited to small samples of patients with no long-term follow up.

**Aims:**

This project aims to utilize intestinal ultrasound assessments obtained at routine time points to determine changes in IUS parameters within 12 months of study enrollment in newly diagnosed children with UC, and their relationship to biochemical markers, clinical assessments, and endoscopic findings.

**Methods:**

Pediatric patients with suspected ulcerative colitis were prospectively enrolled through the Edmonton Pediatric IBD Clinic. Participants underwent IUS, clinical assessments, bloodwork for biomarkers, and fecal calprotectin (FCP) testing at baseline, 1-, 3-, 6-, and 12-month visits, as well as endoscopy at baseline and 6-12 months. In each IUS assessment, the bowel wall thickness (BWT), presence of hyperemia, abnormal haustrations, and fat wrapping was determined in 4 bowel segments (right, transverse, descending, and sigmoid colon). The Pediatric Ulcerative Colitis Activity Index (PUCAI) scores and Ulcerative Colitis Intestinal Ultrasound Scores (UC-IUS) were calculated at each visit.

**Results:**

42 patients (55% male) were included. Median UC-IUS total scores and BWT alone improved in all bowel segments over the study period. For the most affected bowel segment for all patients, median percent reduction of BWT was 39.6%, 34.2%, 33.7%, and 42.5%, at the 1-, 3-, 6-, and 12-month assessment respectively. The biggest decrease in UC-IUS scores and BWT occurred with the initiation of therapy between baseline and 1-month. UC-IUS scores significantly correlated with endoscopy at 12-months, clinical assessments at all time points, and biochemical markers of disease at all time points over the 12-month study period (pampersand:003C0.05). BWT measurement alone also had significant correlation with endoscopy at 12 months; clinical assessment at 1-, 3-, and 6-months; and with biochemical markers at every time point (pampersand:003C0.05). Patients with inflammatory bowel markers (indicated by elevated FCP (ampersand:003E250)) at 12-months were found to have significantly thicker BWT and UC-IUS scores at 3- and 6-months.

**Conclusions:**

BWT and UC-IUS performed well in pediatric patients with newly-diagnosed UC, with UC-IUS scores and BWT measurements alone correlating significantly with endoscopic, clinical, and biochemical disease activity over the 12-month study period. BWT alone performed similarly to total UC-IUS scores. This data suggests that IUS can offer a non-invasive method to monitor long-term pediatric UC disease severity.

**Funding Agencies:**

TRIANGLE, WCHRI, Stollery Children's Hospital Foundation.

